# Oral rehabilitation with implant-supported overdenture (ISO) in four children with ectodermal dysplasia

**DOI:** 10.1186/1746-160X-8-S1-P7

**Published:** 2012-05-25

**Authors:** M Montanari, M Callea, F Battelli, G Corinaldesi, L Sapigni, C Marchetti, G Piana, G Fedele

**Affiliations:** 1 Department of Oral Science, University of Bologna, Italy; 2 Institute for Maternal and Child Health, Trieste, Italy; 3 National Ectodermal Dyplasia Association, Italy

## Introduction

Ectodermal Dysplasias (ED) are a heterogeneous group of inherited disorders characterized by dysplasia of tissues of ectodermal origin. Complete or partial anodontia are the most frequent dental findings. Prosthetic rehabilitation is recommended from functional, esthetic, and psychological points of view. Because of the anatomical abnormalities of existing teeth and alveolar ridges, conventional prosthetic rehabilitation in young patient is often difficult.

## Patients and methods

Four growing patients (age 9 to 11 years) with oligo- or anodontia were prosthetically rehabilitated. Panoramic film and Cone Bean Computerized Tomography were performed and a resin model of mandibular bone was made. Despite a remarkable multi-dimensional atrophy of the alveolar bone, the insertion of two tapered implants was possible. After a submerged healing period of 2 month, the implants were exposed and abutment connection was performed. Implants were connected with an expansion bar that permits mandibular growth and prosthetic retention. A removable prosthesis was constructed with ball attachments. Mandibular growth was followed and evaluated using the expansion guide and cephalometric radiographs.

## Results

Mandibular growth in sagittal and transverse direction had no adverse effects on implant position. The expansion bar permitted the undisturbed growth of the mandible. After 3 years of follow-up, this study showed that ISO may improve oral function, phonesis and esthetics.

## Discussion

The mandibular rotation accompanying growth had not caused a significant problem relative to the angulation of the implants. Implants can be successfully placed, restored and loaded in growing ED patients. The cephalometric analysis supported that ED patients show midface hypoplasia with a class III tendency, which can be avoided by early rehabilitation. Thanks to the good stability and retention of the ISO, patients considered the prostheses as comparable to natural teeth.

